# Strengthening the Capacity of Service Providers to Reduce the Impact of the COVID-19 Pandemic on African, Caribbean, and Black Communities: Protocol for the COVID-19 African, Caribbean, and Black Providers Project 2.0 Implementation and Evaluation

**DOI:** 10.2196/66546

**Published:** 2025-10-14

**Authors:** Josephine Etowa, Hugues Loemba, Liana Bailey, Sanni Yaya, Charles Dabone, Egbe B Etowa, Bishwajit Ghose, Wale Ajiboye, Jane Tyerman, Marian Luctkar-Flude, Jennifer Rayner, Onyenyechukwu Nnorom, Robin Taylor, Goldameir Oneka, Bagnini Kohoun, Wangari Tharao, Haoua Inoua, Ruby Edet, Joseph Kiirya, Ky’okusinga Kirunga, Janet Kemei, Arone Fantaye

**Affiliations:** 1 School of Nursing Faculty of Health Sciences University of Ottawa Ottawa, ON Canada; 2 Montfort Hospital Ottawa, ON Canada; 3 The George Institute for Global Health Imperial College London London United Kingdom; 4 School of Nutrition Sciences Faculty of Health Sciences University of Ottawa Ottawa, ON Canada; 5 Daphne Cockwell School of Nursing Faculty of Community Services Toronto Metropolitan University Toronto, ON Canada; 6 Interdisciplinary School of Health Sciences Faculty of Health Sciences University of Ottawa Ottawa, ON Canada; 7 St Michael’s Hospital Unity Health Toronto Toronto, ON Canada; 8 School of Nursing Faculty of Health Sciences Queen’s University Kingston, ON Canada; 9 Alliance for Healthier Communities Toronto, ON Canada; 10 Dalla Lana School of Public Health University of Toronto Toronto, ON Canada; 11 Ottawa Public Health Ottawa, ON Canada; 12 Canadians of African Descent Health Organization Ottawa, ON Canada; 13 Women’s Health in Women's Hands Community Health Centre Ottawa, ON Canada; 14 AIDS (acquired immunodeficiency syndrome) Committee of Ottawa Ottawa, ON Canada; 15 Somerset West Community Health Centre Ottawa, ON Canada; 16 River Jordan Ministry Ottawa, ON Canada; 17 African Caribbean Council on HIV/AIDS in Ontario Toronto, ON Canada; 18 Department of Mental Health Nursing and Community Wellness MacEwan University Edmonton, AB Canada

**Keywords:** COVID-19, education, health and social service providers, health equity, critical health literacy, critical racial literacy

## Abstract

**Background:**

The COVID-19 pandemic emerged as an unprecedented challenge for health care systems across the world, disproportionately impacting immigrant and racialized populations. Canadian African, Caribbean, and Black (ACB) communities represent some of the populations considered most vulnerable in terms of their susceptibility to health-related risks, receipt of adequate care, and chances of recovery.

**Objective:**

The COVID-19 ACB Providers Project 2.0 aims to strengthen the ability of health care providers to address this community’s COVID-19–related health care needs. Informed by the COVID-19 ACB Providers Project 1.0, a mixed methods study that examined the COVID-19 pandemic’s impact on African, Caribbean, and Black communities in Ontario (Ottawa and Toronto), this second study seeks to develop and implement educational programs in 5 key areas (modules) to strengthen the capacity of service providers (SPs) working with African, Caribbean, and Black populations. These modules (topics) include (1) the COVID-19 pandemic and its impacts on health, (2) social determinants of health and health inequities, (3) critical health literacy, (4) critical racial literacy, and (5) cultural competence and safety.

**Methods:**

An implementation science approach is used to guide the development, implementation, and evaluation of the evidence-informed interventions. The intersectionality lens, socioecological model, and community-based participatory research frameworks are informing the research process. To ensure active stakeholder engagement, the project advisory committee includes 16 African, Caribbean, and Black community members, health providers, and partner agency representatives. Five modules have been developed: 2 web-based simulation games in collaboration with leading simulation experts and 3 nonsimulation modules. The analysis, design, development, implementation, evaluation instructional design method was used as a framework to design and develop the educational modules.

**Results:**

The module development was completed in early 2024, and the training program was officially launched in April 2024 at the University of Ottawa. The synchronous format was delivered until November 2024, and the asynchronous format remained available until the end of May 2025. By January 2025, a total of 322 SPs from 33 organizations had completed training. The focus groups were held in November and December 2024 and surveys were administered between June 2024 and May 2025. Data cleaning will continue until September 2025, analysis is scheduled for September and October 2025, and findings are expected to be submitted for publication in November 2025.

**Conclusions:**

This protocol outlines a scalable, community-informed intervention to build SP capacity in delivering equitable care to African, Caribbean, and Black communities. The findings will inform future health equity training and policy development across Canada and beyond. The results of this study will be disseminated in community workshops, web-based learning platforms, at academic conferences, and in peer-reviewed publications.

**International Registered Report Identifier (IRRID):**

DERR1-10.2196/66546

## Introduction

### Background

The COVID-19 pandemic emerged as an unprecedented challenge for health care systems and has disproportionately affected immigrant and racialized populations globally [1-5]. Canadian African, Caribbean, and Black communities represent some of the populations considered most vulnerable, due to a combination of heightened susceptibility to infection, inadequate receipt of care, poorer recovery outcomes, and death [6,7]. Existing data show that the neighborhoods with the highest proportions (≥25%) of racialized groups (or visible minority) groups experienced a COVID-19 pandemic mortality rate twice as high as neighborhoods with the lowest proportions (<1%) of racialized populations [8].

Social determinants, including racism, income and employment insecurity, housing insecurity, and limited access to health care, are known to account for the disproportionate health risks and differential health outcomes experienced in African, Caribbean, and Black populations [9-13]. In the case of the COVID-19 pandemic, African, Caribbean, and Black individuals are overrepresented in risky frontline and essential jobs with a greater risk of exposure and less access to protections. Excess cases and deaths have been attributed to disproportionately high rates of comorbid conditions (eg, diabetes and hypertension) in African, Caribbean, and Black communities [14]. National surveys indicate that economic impacts have compounded these risks. Black Canadians reported economic vulnerability arising from the COVID-19 pandemic crisis, with 33.2% living in a household reporting difficulty to meet its basic financial commitments in the last 4 weeks [15]. This was almost twice as likely as non–visible minority Canadians (16.6%). The unemployment rate rose more sharply among Black Canadians, by 5.3 percentage points, compared to a 3.7 percentage-point increase among non–visible minority Canadians, which contributed to difficulties in fulfilling housing-related financial obligations [15]. These intersecting inequities reinforce the need for targeted and equity-oriented public health responses.

From a health systems perspective, the consequences of these inequities are far-reaching. The increased burden of the COVID-19 pandemic morbidity and mortality among African, Caribbean, and Black populations contributed not only to individual health harms but has also translated into greater challenges for health systems and for governments through loss of social capital, productive labor force, and erosion of cultural equity [16,17]. Evidence consistently suggests that “one size does not fit all” in terms of the response to public health disasters in populations considered vulnerable [18,19].

It is well documented that African, Caribbean, and Black communities experience multiple and intersecting barriers to accessing appropriate and responsive health services, from institutional discrimination, inadequate training of service providers (SPs), and lack of culturally responsive services in relevant languages to poor representation in health care leadership, research, and decision-making structures [20,21]. Services often fail to reflect the linguistic, cultural, and lived realities of the communities they aim to serve, and consequently, access is limited, distrust grows, and inequities deepen over time.

Numerous scholars have identified the urgent need to build capacity (eg, culturally responsive training) and reduce stigma and paternalism among health providers working with African, Caribbean, and Black communities [22-24]. Central to these recommendations is the widespread acknowledgment that African, Caribbean, and Black communities and scholars must not only be recipients of care but also be actively involved in all aspects of health system responses (eg, prevention, treatment, and outreach-related policy and practices) [24-26].

From 2020 to 2021, the COVID-19 African, Caribbean, and Black Providers Project (CAPP) 1.0 engaged African, Caribbean, and Black community members, health care providers, and system leaders to identify barriers to equitable health care during the pandemic [27]. CAPP 1.0 identified considerable knowledge and capacity gaps among health providers in terms of understanding of African, Caribbean, and Black -specific barriers, antiracism training, cultural responsiveness and safety, and sufficiency of tools to support equitable care. It further generated four priority areas for SPs to meet the needs of African, Caribbean, and Black communities: (1) transform the health system’s capacity to deliver anti-racist care; (2) strengthen the African, Caribbean, and Black community’s capacity for authentic participation in health policy, practice research, education, and leadership; (3) create programs and policies that foster disaggregated race-based data collection, governance, and develop strategic partnerships; and (4) create accountability measures for anti-racist healthcare for African, Caribbean and Black people within all levels of the healthcare system.

In response to the urgency of capacity building, the current phase, CAPP 2.0, directly builds on those findings and targets these priority areas by creating and evaluating web-based capacity-building interventions to address the key gaps from CAPP 1.0. The education sector has adopted and used webinars more extensively in the 21st century to create engaging learning resources, particularly with the onset of the COVID-19 pandemic [28]. The term “webinar” refers to communication over the internet between 2 or more participants using audio, video, and activities, such as polls and quizzes, with the possibility to allocate participants into breakout rooms to work together on a topic or solve a problem [29]. Webinars have been used both synchronously and asynchronously as engaging learning formats. In synchronous formats, learners participate together at a predefined time, while in asynchronous formats, they may engage in a webinar independently at any time. In a recent systematic review, Gegenfurtner and Ebner [30] found that synchronous webinars were slightly more effective than control conditions (web-based asynchronous learning management systems and offline face-to-face classroom instruction), but these differences were trivial in size. Moreover, little research to date has examined the implementation and impact of innovative web-based strategies to address professional capacity-building needs to serve African, Caribbean, and Black communities.

### Objectives

The overarching aim of the CAPP 2.0 project is to accelerate the use of high-quality, real-time evidence (collected on the contextual vulnerability and challenges experienced by African, Caribbean, and Black communities) to codevelop, implement, and evaluate community-driven solutions that confront structural and systemic inequities in health care. To achieve this aim, the project is guided by two core goals: (1) to develop and implement tailored webinar-based training modules to strengthen provider capacity and responsiveness to African, Caribbean, and Black health needs and deliver culturally responsive and antiracist care and (2) to evaluate the uptake, engagement, and effectiveness of these interventions for improving provider capacity. For the purpose of this study, we use “service providers” as an umbrella term throughout the manuscript to refer collectively to health care professionals (including physicians, nurses, and allied health staff), students in training to become health professionals, public health professionals. professors, social workers, and staff members (eg, managers) from community-based organizations. To guide the design, delivery, and evaluation of the training modules, the CAPP 2.0 project draws on community-based participatory research (CBPR) principles [31] and established implementation science frameworks. It adopts an intersectionality lens [32] to account for the overlapping structural barriers that affect African, Caribbean, and Black health outcomes and is informed by the socioecological model (SEM) [33] which highlights the interplay between individual, interpersonal, community, organizational, and systemic factors that influence health access. The Consolidated Framework for Implementation Research (CFIR) provides further structure to assess how the intervention design, setting characteristics, and external policies influence implementation [34]. To support the overarching aim and project goals, the CAPP 2.0 project has four specific objectives: (1) engage African, Caribbean, and Black communities and key stakeholder groups in the codevelopment of community-driven strategies to reduce the burden in these communities during and after the pandemic; (2) develop and implement innovative evidence-based modular health educational interventions (ie, critical health and racial literacy modules) to address the gaps identified in CAPP 1.0; (3) assess the reach, uptake and effectiveness of the synchronous and asynchronous webinar formats to enhance health care SPs’ ability to provide effective health care for African, Caribbean, and Black people; and (4) improve evidence-based communication among health stakeholder groups (ie, health planners, health providers, and African, Caribbean, and Black communities) to reduce COVID-19–related health inequities.

The flexible formats, context-specific and community-rooted training content are expected to promote SP engagement. Participating providers are expected to demonstrate increased knowledge of critical racial and health literacy principles, improved confidence in delivering culturally safe care, and a greater alignment between service delivery and the needs of African, Caribbean, and Black communities. Ultimately, effective webinar-based education that is codeveloped and grounded in the lived experiences of their communities has the potential to mitigate systemic barriers and push for health equity reforms.

## Methods

### Conceptual Frameworks

CBPR frameworks and an implementation science approach are being used to guide the development, implementation, and evaluation of the evidence-informed interventions to transform the health system’s capacity to address structural inequities and anti-Black racism in health care. CBPR [31] is being used to involve African, Caribbean, and Black communities in the co-design and implementation of interventions, practices, and policy tools. Community members are to participate and take ownership of the process and cocreate innovative solutions that reflect the lived experiences of African, Caribbean, and Black communities, in partnership with other stakeholders and decision makers.

In addition, the intersectionality lens and SEM frameworks are informing the design and research process. Intersectionality is an analytical tool used in equity work to understand and interpret the complexity of the world around us [32]. It emphasizes that health issues are influenced by broader social factors and do not occur in isolation, but rather these factors intersect and mutually enhance their negative impacts on health outcomes [35]. In this project, it provides a critical lens to understand how overlapping social identities and systems of oppression contribute to the health inequities that the African, Caribbean, and Black population experiences. This lens informs all project stages and promotes reflexivity and equity-centered interpretation throughout the research process. SEM identifies a vast array of layered macro-, meso-, and microlevel factors [33] to consider when addressing health inequities, including COVID-19 infection and its impact among racialized populations. In this project, SEM is being operationalized to contextualize the multiple layers of influence (individual, interpersonal, community, organizational, and policy) and inform a more holistic intervention. The Analysis, Design, Development, Implementation, Evaluation instructional design model [36] helped translate these complex insights into actionable educational interventions and provides a practical and systematic model for testing and refining webinar modules responsive to community-identified needs.

Finally, the CFIR provides a comprehensive structure to assess multidimensional domains associated with effective implementation (ie, intervention characteristics, outer setting, inner setting, characteristics of individuals, and process) [34]. The focus of this project is on constructs of the outer setting, such as external policy and incentives, and constructs of the inner setting, such as climate and leadership. The CFIR framework is a good fit for CAPP 2.0 specifically given its extensive use in community-based programs and interventions as well as its capacity to tailor interventions according to the research objectives. A variety of tools are being developed for the evaluation, including website analytics, preintervention and postintervention surveys, and follow-up interviews with providers and agencies.

Together, these conceptual frameworks are being used synergistically to garner community involvement and participatory development, frame a more nuanced understanding of overlapping identities and structural inequities, develop and implement a multilevel and holistic intervention, and guide a rigorous evaluation with an explicit equity focus. In addition, this triangulation ensures that the intervention is more culturally relevant, contextually sensitive, practically feasible, and evaluable within complex health systems.

### The Research Process

#### Overview

This 3-year research project is integrative and organized in phases. Phase 1 focuses on preplanning and module development—stakeholder engagement and knowledge mobilization tools development (year 1) to raise awareness among African, Caribbean, and Black people about the social determinants of COVID-19 pandemic vulnerabilities and to set the stage for the learning modules implementation and uptake; phase 2 involves innovative module development and implementation (year 2) for SPs’ collective empowerment and capacity building to build requisite skills, abilities and critical perspectives for effective COVID-19 pandemic and health equity responses among African, Caribbean, and Black communities; and phase 3 features module implementation and evaluation as well as other knowledge mobilization activities (year 3) to mobilize providers, African, Caribbean, and Black people and other stakeholders in the collaborative development and implementation of evidence-informed programming, research and policy to address COVID-19–related health inequities in African, Caribbean, and Black communities.

#### Phase 1

To ensure active stakeholder engagement, a project advisory committee (PAC) has been formed, comprising 16 African, Caribbean, and Black community members, health providers, partner agency representatives, and other stakeholder groups. The PAC meets quarterly. The development of the web-based learning intervention was led by JE and assisted by the PAC and high-impact field-based interventions laboratory. The intervention consists of a series of five web-based educational modules for SPs aimed at (1) social determinants of health and health inequities, (2) the COVID-19 pandemic and its impacts on health, (3) critical health literacy, (4) critical racial literacy, and (5) cultural competency and safety. These themes were rigorously reviewed and prioritized through iterative consultations with the PAC and a stakeholder engagement event facilitated by the CO-CREATH Lab. The 5 themes were selected based on a comprehensive analysis of findings from the CAPP 1.0 project, which identified critical knowledge and practice gaps among SPs working with African, Caribbean, and Black communities. Specifically, quantitative and qualitative data from CAPP 1.0 revealed a lack of awareness and understanding of the barriers faced by African, Caribbean, and Black clients in relation to health equity, culturally responsive care, and the impacts of the COVID-19 pandemic. These themes were further validated and refined through consultations with the PAC and through a stakeholder engagement event involving African, Caribbean, and Black community members, researchers, SPs, and decision makers facilitated by the CO-CREATH Lab. Each theme is operationalized into individual modules with clearly defined learning objectives that directly target the identified provider competency gaps.

These modules are also grounded in current behavioral science principles and insights, such as formatting messages in clear and easy-to-understand language, using social media platforms commonly used by African, Caribbean, and Black communities, using compelling stories from the community, making educational events social, and using evidenced-informed messages delivered by trusted messengers, such as African, Caribbean, and Black community health professionals. The instructional design process was guided by the analysis, design, development, implementation, evaluation model [36] to systematically plan, test, and refine the modular context based on stakeholder feedback and implementation feasibility.

Each theme has been integrated into the educational modules using a combination of instructional strategies, including narrative-based learning, community-informed scenarios, and virtual simulation (VS) pedagogy. These instructional strategies were selected to support the intended learning outcomes in a manner that maximizes engagement, cultural relevance, and opportunities for critical reflection. For example, the Canadian Alliance of Nurse Educators using Simulation virtual experience templates developed for CAPP 2.0 include character sketches and real-life scenarios drawn from CAPP 1.0 participant quotations. This enabled each module to reflect the lived experiences and contextual realities of African, Caribbean, and Black communities. An e-learning expert and instructional design consultants were contracted to guide the development of the nonsimulation modules for themes 1 and 2.

The other 3 modules for themes 3, 4, and 5 are VS educational modules. VS educational modules are innovative and accessible resources that provide effective instruction and critical thinking in health care education, comparable with or exceeding outcomes associated with other simulation modalities or teaching methods [37-39]. The benefits of VS pedagogy were especially highlighted during the COVID-19 pandemic when most health care education had to shift to virtual formats. Learners report that VS is an engaging and enjoyable educational strategy that is easy to use and contributes to their applied learning [40]. Additional benefits include the ability to place all learners in decision-making roles, to increase accessibility to larger groups of learners, to provide instant and evidence-based feedback, to increase flexibility in self-pacing, and to have the potential to create a more psychologically safe learning environment [41]. Authors ML-F and JT are VS experts who supported the creation of these simulation modules using the Canadian Alliance of Nurse Educators using Simulation VS design process [42]. The contracted e-learning specialist oversaw the development of an accessible website to host the 5 learning modules.

#### Phase 2

Two different (synchronous and asynchronous) formats have been used and are being tested for the implementation of capacity-building activities among SPs. The first format (synchronous) primarily targets health providers such as physicians and nurses in 5 prespecified practice settings (ie, Ottawa Public Health, Somerset West community health center, Southeast Ottawa community health center, AIDS Committee of Ottawa, and the African Caribbean Council on HIV in Ontario). In each setting, the research team is working with local health partners to engage and deliver training to at least 75% of agency staff through internal communications, staff meetings, and direct outreach by organizational leaders.

**Figure 1:**
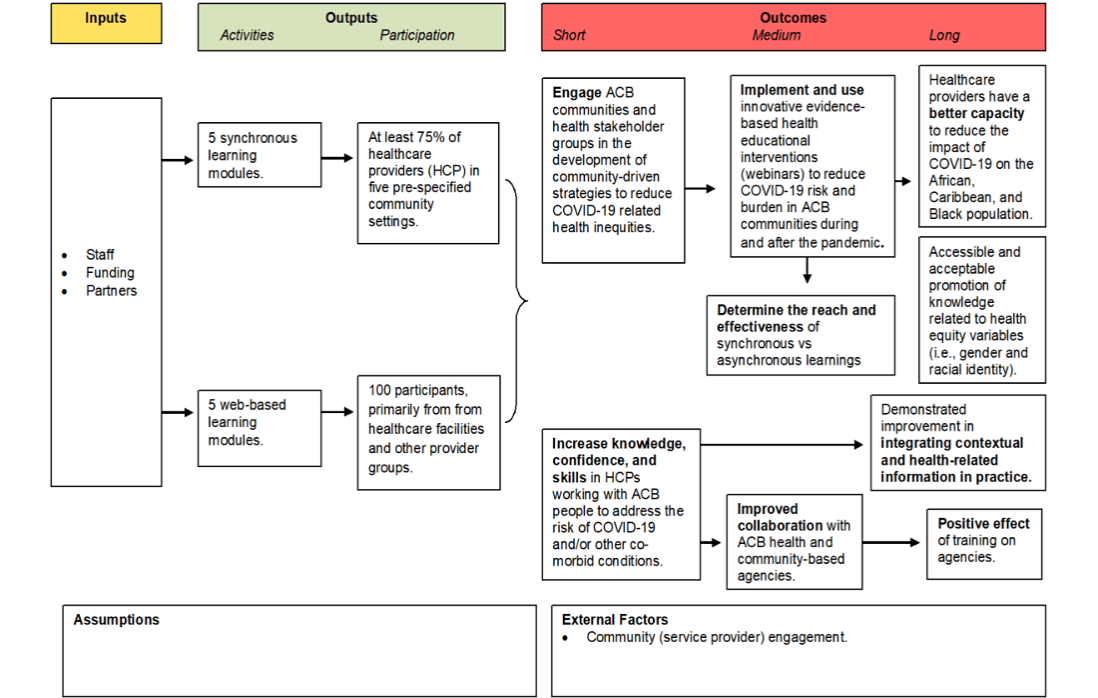
COVID-19 African, Caribbean, and Black Providers Project 2 logic model. ACB: African, Caribbean, and Black.

SPs more broadly are also targeted to engage in the asynchronous web-based modules housed in a website developed for this project. Recruitment has been initiated through professional networks, health care institutions, and partner organizations across Ontario. Promotional materials, such as flyers, email invitations, and social media posts were distributed to reach a wide range of SPs, including physicians, nurses, allied health professionals, and students. The website and web-based modules have been primarily promoted to health care facilities (eg, primary care, hospitals, long-term care, and community organizations) and provider groups (eg, physicians, nurses, and allied health staff). The aim was to reach a minimum of 100 individual SPs with our broader outreach activities. This target sample size was set based on the reach experiences from CAPP 1.0, using similar recruitment pathways. In addition, it reflects a balance of recruitment feasibility (limitations of provider time and capacity), diversity, and representation from organizations across primary care, public health, and community-based services in Ontario. This minimum target would enable us to compare implementation experiences, engagement patterns (synchronous vs asynchronous), and learning outcomes across sectors and learning formats, while also supporting adequate variation for subgroup analysis (eg, by profession, delivery format, or organizational setting). Figure 1 illustrates the logic model which is being used to track project indicators throughout.

#### Phase 3

A comprehensive mixed methods evaluation is being continuously conducted to assess the project’s activities and impacts, including implementation outputs, short-term and long-term outcomes (as seen in the logic model in Figure 1). The preprogram and postprogram module surveys are linked to each module’s learning objectives and must be completed by all participants to track changes in knowledge, confidence, and the perceived ability to deliver culturally responsive and equity-focused care to African, Caribbean, and Black people. All recruited participants have to complete the pretraining survey before participating in the program. After completing the program, they are directed to the posttraining survey (on Survey Monkey).

Participants recruited for the postprogram focus group discussions (FGDs) are purposively sampled from among those who complete the training modules. Invitations are sent out by email for representations across roles, including primarily health care professionals. Participation is voluntary, and informed consent is obtained before participation. The focus groups are being used to evaluate the reach, implementation challenges or successes, and effectiveness of the training modules. Survey data will be analyzed using descriptive statistics, including measures of central tendency (eg, mean) and variability (eg, range), to assess program reach, participant satisfaction, and self-reported changes in knowledge and capacity. Website analytics will also be examined using descriptive statistics to examine metrics, such as the number of users, time spent per module, and completion rates. All quantitative analyses will be conducted using statistical software, such as SPSS (IBM Corp) or Microsoft Excel. FGD recordings will be transcribed and coded inductively using Microsoft Excel or NVivo (Lumivero). Thematic analysis will be conducted on the FGD data. Multiple members of the research team will be involved in the generation, review, and validation of codes and themes. The final themes will be refined collaboratively for better alignment with the study’s theoretical frameworks and objectives, and to provide a comprehensive understanding of participants’ training experiences and feedback.

The implementation research and evaluation is being informed by CFIR, with consideration of its 5 domains: intervention characteristics, outer setting, inner setting, characteristics of the individuals involved, and the process of implementation [34]. Furthermore, this project focuses on the characteristics of the inner and outer settings that influence implementation (eg, structural characteristics, policies, and implementation climate). Specifically, this project is interested in learning whether SPs who participate in setting-specific training using the web-based modules are more likely to benefit from the training and engage in quality improvement and system transformation initiatives in their agencies than SPs who complete the web-based modules independently.

Together, the preintervention and postintervention survey items, focus group topics, completion metrics, and the CFIR-guided evaluation comprise an integrated evaluation framework to assess both instructional effectiveness and implementation fidelity in relation to each module’s objectives. This integrated evaluation approach assesses not only learning outcomes but also the utility and sustainability of the intervention within real-world health care settings. The expected short-term outcome for SPs includes increased knowledge, confidence, and skills in working with African, Caribbean, and Black people to address the risk of COVID-19 infection and other comorbid conditions. Long-term outcomes include a demonstrated improvement in integrating contextual and health-related information in practice, a positive effect of training on agencies, and improved collaboration with African, Caribbean, and Black health and community-based agencies.

### Rigor and Dissemination

To establish the trustworthiness and scientific rigor of this research, the research process will be guided by the framework for assessing the quality of qualitative research postulated by Lincoln and Guba [42]; that is, credibility, transferability, confirmability, and dependability. Credibility refers to the confidence readers have in the analytical and interpretive processes and findings. In this research, transparent analytical steps are identified and informed by established qualitative research principles. Transcripts will be carefully verified and checked. In the analysis process, attention will be paid to misrepresentation of the evaluation component, FGD data. We will interrogate the data until data saturation is reached. Furthermore, the research team is trained in the conduct of ethical and sensitive research with this study populations. Transferability is the extent to which the study results might be relevant to similar populations that have similar characteristics. Transferability is dependent on “thick description,” which in this work will be the detailed and precise narrative that will be constructed following the comprehensive analysis of the evaluation data. The narrative description will provide sufficient detail for others to make a judgment on the quality of the results. Confirmability will be achieved by reflexive team and PAC meetings. Dependability will be achieved by the establishment of a clear audit trail and accurate documentation of the research processes and procedures, including the analytical process, field notes, digital recordings, and transcripts.

### Ethical Considerations

The ethics approval was obtained (July 2023) from the Research Ethics Boards of the University of Ottawa (H-01-23-8069), and operational site permission will be obtained where necessary before commencing research.

Voluntary written informed consent will be obtained from all participants. As part of the consenting process, participants will be assured that they do not have to answer every demographic or FGD question, they can choose to be recorded on audio (or not), and they can withdraw at any time. They will especially be informed that their nonconsent will not affect their employment conditions.

The principles of confidentiality and anonymity will be observed at all times, including during data collection, analysis, and in the storage of research materials. Research materials will be securely stored on password-protected digital platforms or in locked physical locations. If instances of distress are encountered, participants will be offered information about counseling support services. Participants will receive a gift card (CAD $25 or $30 [US $18 or $22]) compensation for their time and contributions. The posttraining survey and FGD will be performed on a secured digital platform (SurveyMonkey) or in-person location at the University of Ottawa or a partner agency.

No identifiable images or personal data will be included in the manuscript or supplementary materials. If any such materials are used in future dissemination, explicit written consent will be obtained and documented.

## Results

The CAPP 2.0 training initiative was developed by the CO-CREATH Lab between 2023 and early 2024 as part of the CAPP 2.0 project. The training program officially launched in April 2024 at the University of Ottawa. The asynchronous sessions were delivered until November 2024, while the asynchronous training was available until the end of May 2025. So far, 322 SPs from 33 organizations have participated in the training program. Mixed methods evaluations involving surveys and FGDs are underway to explore participants’ feedback, experiences, perceptions, and self-reported changes in knowledge and capacity of critical areas. Specifically, four focus groups were conducted in November and December 2024, while the surveys were administered between June 2024 and May 2025. Cleaning of the data collected since January 2025 will continue in September 2025, and analysis of our mixed-method data will be completed in September and October 2025. The findings from the program evaluation are expected to be submitted for publication in November 2025.

## Discussion

### Anticipated Findings

This protocol outlines the plans to develop, implement, and evaluate CAPP 2.0, designed to strengthen the capacity of SPs to deliver equitable care to African, Caribbean, and Black communities in Ontario during and beyond the COVID-19 pandemic. The anticipated outcomes include an increase in SP knowledge, confidence, and skills to address barriers to health care access, particularly through the use of culturally responsive, community-informed web-based learning modules. Accessible resources and practice and policy tools will be developed to enable health policy makers and planners throughout the province to meet the health care needs of African, Caribbean, and Black communities currently and after the pandemic. The widespread impact of the COVID-19 pandemic demands that previously considered local concerns now become global concerns. Provincial collaboration, such as the one in this project, will bring different research perspectives to bear on the issue of the COVID-19 pandemic and health service provision from the perspective of African, Caribbean, and Black people in Ontario. Specifically, this project will promote and strengthen knowledge exchange and links to providers, knowledge users, and researchers across Ontario, with the potential for national scale-up. It will facilitate exposure to innovative ideas and approaches. It will provide opportunities for redesigning and identifying promising models for SP capacity building, informed by critical and diverse perspectives. While there are no quick solutions, sustained gains in providers’ COVID-19–related health equity capacity, such as the one that would be provided by this project, will successfully harness health care providers who can address critical postpandemic health priorities.

Moreover, the co-designed, community-involved, and evidence-based model developed through this initiative will provide a scalable and adaptable model or framework to help address health inequities. The model crucially centers the lived experiences of African, Caribbean, and Black communities and integrates VS pedagogy, and in turn informs future interventions across Canada and globally, especially in contexts where racialized populations face systemic and structural barriers to equitable health care. The findings and tools generated through this project can potentially influence broader health policy, inform culturally responsive training initiatives and standards, and contribute to public health efforts to promote health equity.

### Strengths and Limitations

Strengths of this protocol include its grounding in CBPR, intersectionality, its use of implementation science frameworks, VS pedagogy, and its emphasis on cultural competency and safety. The co-designed model centers the lived experiences of African, Caribbean, and Black communities and produces provider training tools and potential practice and policy-informing resources that can support training and health equity reform across sectors. A limitation is that the evolving nature of the pandemic and health system priorities may affect module uptake or relevance over time. While the uptake may be affected by the pandemic priorities, the modular design and flexible delivery formats increase its potential for relevance, scalability, and sustainability.

### Conclusions

The tools, approaches, and findings from CAPP 2.0 may inform culturally responsive training policies and contribute to broader public health and systems change efforts that intend to combat structural and systemic inequities. Consequently, the CAPP 2.0 initiative offers a scalable and adaptable model that could be implemented across Canada and potentially adapted globally in settings where racialized populations face systemic and structural barriers to care. In terms of future directions, there is a need to adapt the model for other marginalized populations, as well as longitudinal assessments of impacts on provider behavior and patient outcomes. The dissemination plan includes community workshops, academic presentations, peer-reviewed publications, and an open-access web-based learning platform for broad accessibility and impact.

## Abbreviations

CAPP: COVID-19 African, Caribbean, and Black Providers Project
CBPR: community-based participatory research
CFIR: Consolidated Framework for Implementation Research
FGD: focus group discussion
PAC: project advisory committee
SEM: socioecological model
SP: service provider
VS: virtual simulation
